# Polymer Colloids: Current Challenges, Emerging Applications, and New Developments

**DOI:** 10.1021/acs.macromol.3c00108

**Published:** 2023-03-23

**Authors:** Miren Aguirre, Nicholas Ballard, Edurne Gonzalez, Shaghayegh Hamzehlou, Haritz Sardon, Marcelo Calderon, Maria Paulis, Radmila Tomovska, Damien Dupin, Ren H. Bean, Timothy E. Long, Jose R. Leiza, José M. Asua

**Affiliations:** † POLYMAT, Kimika Fakultatea, University of the Basque Country UPV/EHU, Joxe Mari Korta Zentroa, Tolosa Hiribidea 72, 20018 Donostia-San Sebastian, Spain; ‡ IKERBASQUE, Basque Foundation for Science, Plaza Euskadi 5, 48009 Bilbao, Spain; § CIDETEC, Parque Científico y Tecnológico de Gipuzkoa, P° Miramón 196, 20014 Donostia-San Sebastian, Spain; ∥ Biodesign Institute, Center for Sustainable Macromolecular Materials and Manufacturing (SM3), School of Molecular Sciences, Arizona State University, Tempe, Arizona 85281, United States

## Abstract

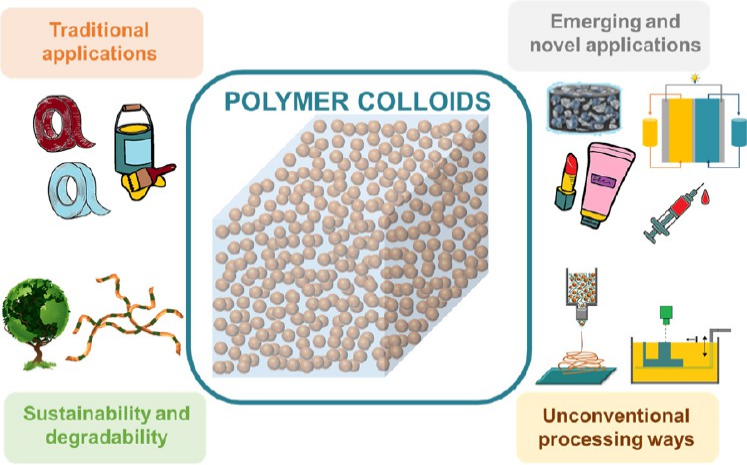

Polymer colloids are complex materials that have the potential to be used in a vast array of applications. One of the main reasons for their continued growth in commercial use is the water-based emulsion polymerization process through which they are generally synthesized. This technique is not only highly efficient from an industrial point of view but also extremely versatile and permits the large-scale production of colloidal particles with controllable properties. In this perspective, we seek to highlight the central challenges in the synthesis and use of polymer colloids, with respect to both existing and emerging applications. We first address the challenges in the current production and application of polymer colloids, with a particular focus on the transition toward sustainable feedstocks and reduced environmental impact in their primary commercial applications. Later, we highlight the features that allow novel polymer colloids to be designed and applied in emerging application areas. Finally, we present recent approaches that have used the unique colloidal nature in unconventional processing techniques.

## 1 Introduction and Motivation

Polymer colloids, often referred to as waterborne polymer dispersions or emulsion polymers, are specialty polymers that are used in a broad range of products. Notable high tonnage applications include coatings, adhesives, additives for paper, textiles, leather and construction materials, impact modifiers for plastics, and binders for nonwoven fabrics. Other products are directed toward high added value applications such as cosmetics and health care.^1,2^

**Figure 1:**
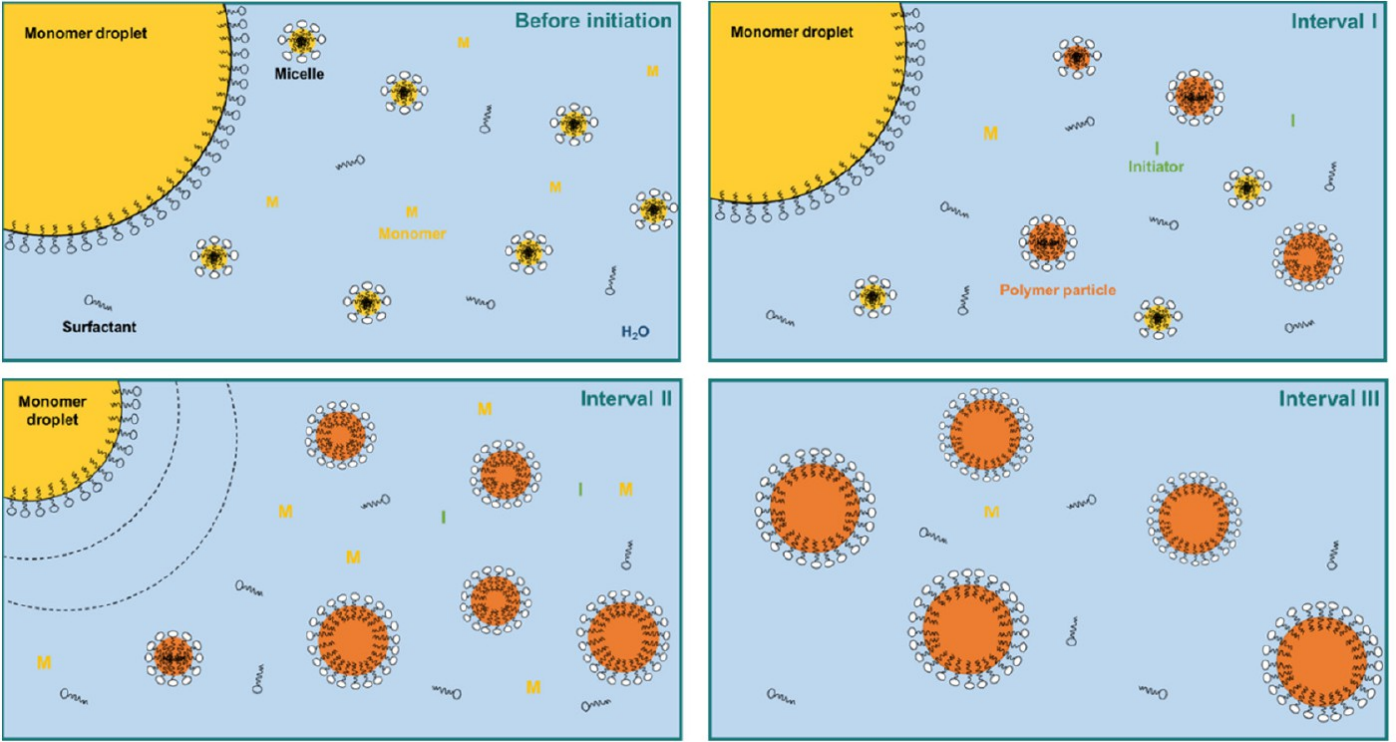
Schematic representation of an emulsion polymerization process.

The main synthetic technique for the production of polymer colloids is emulsion polymerization,^3,4^ which is a complex multiphase polymerization process (see Figure 1). In an emulsion polymerization, an initial dispersion of micrometer-sized monomer droplets in water is converted to a latex consisting of nanometer-sized polymer particles. After close to 100 years of research, this technique now offers excellent control over properties of the polymeric (molar mass, macromolecular architecture, etc.) and colloidal (particle size distribution, particle morphology, etc.) properties of the final emulsion polymer. Aside from the ability to generate complex structures, the development of emulsion polymerization as the primary route to synthesize colloidal polymers has been mainly due to three factors. The first is directly related to environmental concerns and new regulations that have mandated the substitution of solvent-based products by waterborne materials. This has been particularly relevant in the growth seen in the market for emulsion polymers in the coatings and adhesives sectors in the last few decades.^5−7^ As will be highlighted in this perspective, the drive toward even lower volatile organic content and more sustainable products is a major societal challenge that can only be resolved by improving our fundamental knowledge. The second factor relates to the versatility of the polymerization process for synthesizing colloidal polymers with unique properties. The ability to synthesize complex structures on a large scale has not only been helpful to overcome a number of technical issues related to the major markets of emulsion polymers but also should help in the application of colloidal polymers in several new areas. The final factor that has contributed to the growth of emulsion polymerization is related to the scalability of the process; compartmentalization of radicals in nanosized particles leads to high rates of polymerization and high molar mass polymers that can be handled without any major viscosity issues,^8^ while the use of water as dispersing media allows for efficient heat removal during polymerization.

In addition to emulsion polymerization, which remains the most popular technique for synthesis of colloidal polymers at the industrial scale, a number of other polymerization processes in dispersed media are also used for some speciality products. Thus, polymers that are completely soluble in water like polyacrylamide (used for soil conditioning and enhanced oil recovery)^9^ and poly(acrylic acid) (highly absorbent material used in disposable diapers and napkins)^10^ are produced by inverse emulsion polymerization. Miniemulsion polymerization is used when water-insoluble materials such as preformed polymers, highly hydrophobic monomers, and inorganic particles should be incorporated in the polymer particles.^11^ This technique is particularly useful for implementing reversible deactivation radical polymerization (RDRP).^12,13^ In addition, dispersion polymerization is used to produce micron-size polymer dispersions that find applications for toners and packing materials for chromatography.^14^ Inverse microemulsion polymerization is used mainly to synthesize high molar mass cationic polymers that find use as flocculants in wastewater treatment.^15^ Finally, suspension polymerization is widely used for the production of poly(vinyl chloride) and expandable polystyrene among many other commodity polymers.^16^ However, in this process, polymerization occurs in large monomer droplets, where there is no compartmentalization of radicals, and therefore, the characteristics of the polymers are similar to those obtained in bulk. In addition, it produces polymer particles which lie outside of the colloidal size range and will not be discussed further in this perspective.

One reason for the commercial success of colloidal polymers is their versatility and the ability to tune the properties of the final material during the synthetic process. For instance, the final properties of a given latex system can be influenced (and controlled) by the polymer composition, chemical composition distribution, molar mass distribution, polymer architecture, grafting, cross-linking, particle size distribution, particle morphology, and particle surface composition. In certain applications, some of these properties take precedent over others. For example, when applied as pressure sensitive adhesives, the molar mass distribution and polymer architecture are of primary importance as they dictate the rheological response of the adhesive. In contrast, for many emulsion polymers used in coatings, particle morphology is the primary parameter used to control mechanical properties and the molar mass distribution is of secondary importance. As will be detailed below, this range of structural features allows polymer colloids to be potentially used in a huge range of different applications aside from the current commercial applications. On the other hand, this complexity also represents a challenge. As for a given application, several (often conflicting) properties are needed. For example, an ideal pressure sensitive adhesive should present good tack and high peel and shear resistance. Unfortunately, many of the parameters that result in improved shear resistance, e.g., by increasing the molar mass and/or the cross-linking density of the polymer, result in deterioration of the tack.^17^

Although more than 20 million (wet) tons of colloidal polymers are produced annually,^18^ there is still much to learn about colloidal polymers and the process by which they are produced, as well as their potential use in new application areas. In this perspective, we will attempt to summarize our view of the current challenges in the synthesis and use of polymer colloids and future directions for research. First, we will outline the unresolved issues that remain with regard to the fundamental understanding of the emulsion polymerization process as well as the behavior of latexes in the main commercial application areas. Long-standing unsolved challenges^19^ include complete knowledge of the mechanisms controlling the polymerization, online control of the process to cope with run-to-run irreproducibility, and understanding the mechanism through which the polymer dispersion is transformed into a film. Following this, we direct our attention to the transition toward more sustainable synthesis of polymer colloids and options to improve their end-of-life management. Subsequently, we discuss more recent developments on the synthesis polymer colloids with new functionalities and the expansion into new areas such as cosmetics and health care, energy, CO_2_ capture. Finally, we highlight alternative processing methods for colloidal polymers such as electrospinning and additive manufacturing that have recently appeared.

## 2 Fundamental Challenges in the Production of Emulsion Polymers

### 2.1 Unsolved Issues

Although emulsion polymerization has been conducted on an industrial scale for close to 100 years, there are a number of mechanistic details that are yet to be resolved.^20,21^ In many cases, this lack of knowledge represents a major issue and results in researchers resorting to trial-and-error approaches to improve product performance. Early work clearly established that the central locus of polymerization in emulsion polymerization is the polymer particles that are formed during the process.^22,23^ The polymerization rate in the polymer particles, where the overwhelming majority of polymerization occurs, is given by a deceptively simple equation:
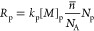
1where *k*_p_ is the propagation rate constant, [*M*]_p_ the monomer concentration in the polymer particles, *n̅* the average number of radicals per particle, *N*_A_ Avogadro’s number, and *N*_p_ the number of particles.

The rate coefficients for propagation for many commonly used monomers have been benchmarked by an IUPAC working party.^24−28^ Methods to estimate the monomer concentration in the polymer particles are available, although partitioning of hydrophilic monomers is sometimes uncertain.^29^*n̅* is determined by the interplay between radical entry and exit and termination.^30^ Quite a few models for radical entry^31−34^ and exit^35,36^ are available. However, in spite of the considerable effort devoted to experimentally determine these rate coefficients,^37−42^ investigations have not been conclusive.^16^ Actually, the existing models could not explain the competitive growth of particles of different size.^36^ The complexities of chain growth at low degrees of polymerization, especially in polar media where the rate coefficient of propagation can vary by an order of magnitude^43^ and the nature of entry for copolymerization systems with monomers of different hydrophobicities,^44−46^ are some of the reasons for the failure of the current models.

**Figure 2:**
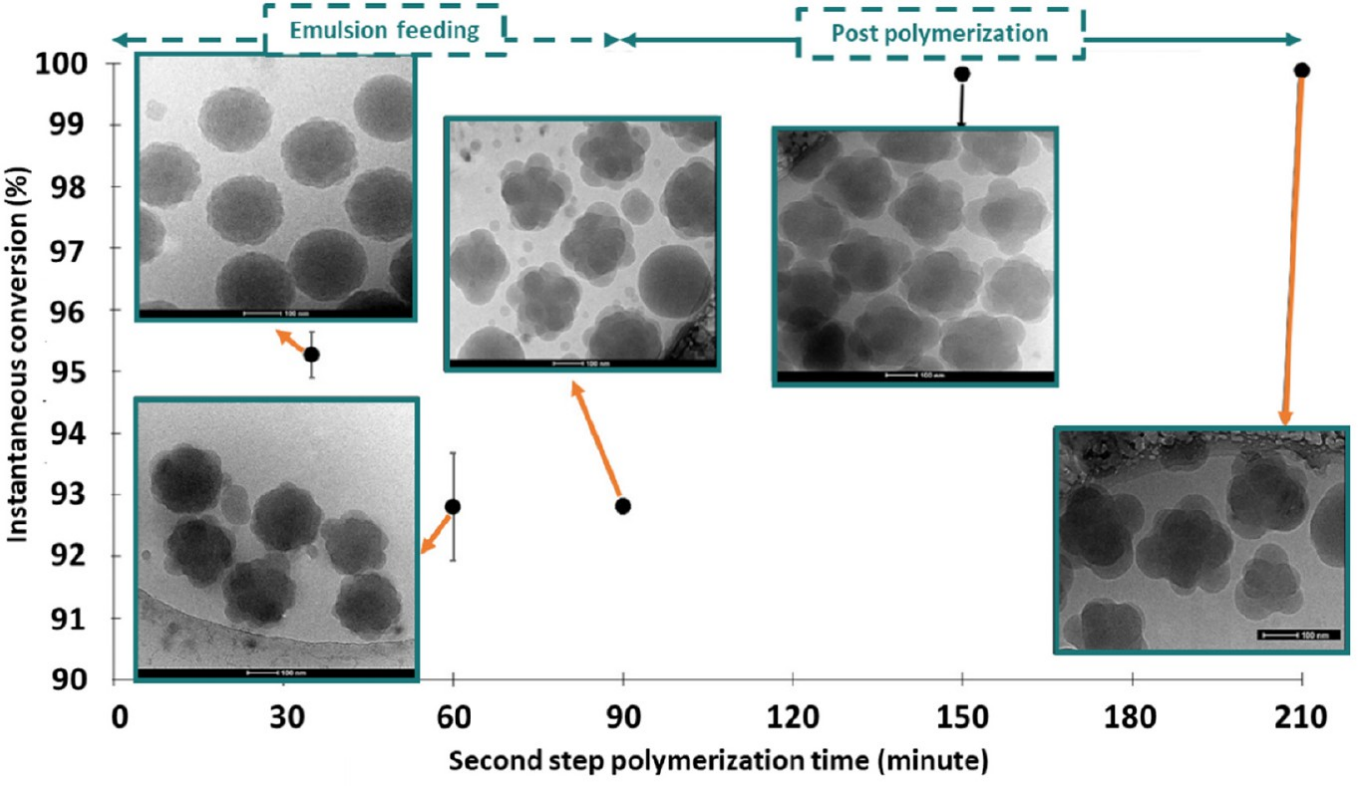
Evolution of the particle morphology in a seeded semicontinuous emulsion polymerization. Reprinted from ref (48). Copyright 2019 American Chemical Society.

Radical termination is also affected by uncertainties. In addition to the expected challenges due to the chain-length-dependent termination^47^ and the diffusion-controlled termination (gel effect), there is a not often discussed issue that affects the compartmentalization of radicals within the particles that may occur in hybrid latexes. Thus, Figure 2 presents the evolution of the particle morphology in the seeded semicontinuous emulsion copolymerization of styrene, butyl acrylate, acrylamide, and acrylic acid.^48^ The composition of the seed was different and this led to phase segregation. It can be seen that a multitude of clusters of the second stage polymer is formed at the surface of the seed and that they grow over time. Even if the clusters are in the same particle, radicals in different clusters cannot terminate between them, namely, there is an additional radical compartmentalization that is not currently accounted for. This problem does not only affect hybrid latexes, as surface anchoring of the entering charged radicals may also lead to an additional compartmentalization effect within the particle.

**Figure 3:**
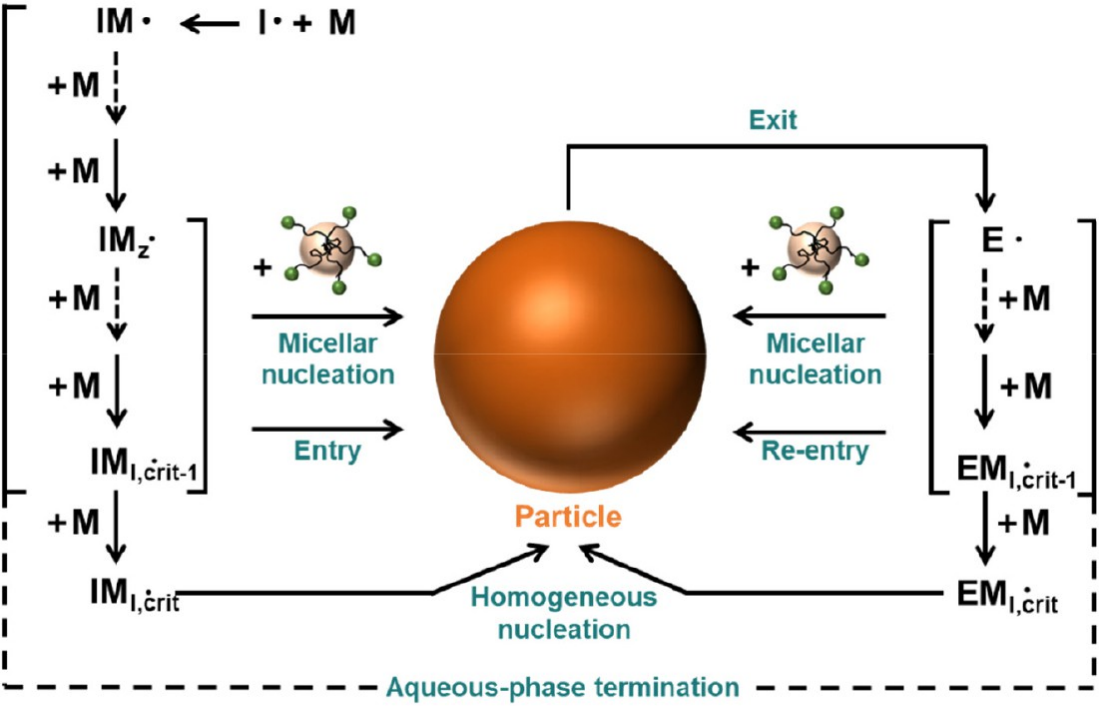
Scheme of the aqueous phase and phase-transfer events considered in the present work. Adapted from ref (55). Copyright 2009 American Chemical Society.

Linked to this is the issue of particle formation itself and the evolution of particle size distribution during the reaction. Radicals generated in the aqueous phase can either enter pre-existing particles or can nucleate new particles by one of two distinct mechanisms (see Figure 3). In the absence of surfactant or pre-existing seed particles, chain growth can continue in the aqueous phase until a critical chain length at which the oligomer is no longer soluble in aqueous media and forms a primary particle.^49,50^ Subsequent coagulation of these primary particles can occur until a stable particle is formed that will then compete for radical capture.^51^ In the case that surfactant micelles are present, in addition to homogeneous nucleation, a competitive nucleation process known as hetereogeneous nucleation can take place through the entry of an oligomeric radical into a micelle to form a primary particle.^23^ Mathematical modeling of these kinds of nucleation processes has some limited success in explaining the particle size distribution but requires information about propagation behavior of oligomeric chains as well as the nucleation and coagulation behavior after the critical chain length is reached.^52,53^ As the relevant kinetic and thermodynamic parameters required are not readily available, especially for the more complex practical situations of interest, such models cannot really be robustly tested at present and have limited predictive capacity for complex copolymerization systems. Furthermore, even if the nucleation process can be well-defined, due to our poor understanding of the nature of the radical entry/exit process, the subsequent competitive growth between particles with different particle sizes makes it very challenging to predict the evolution of particle size distribution with time using the currently available models.^54^

Perhaps the central issue related to the understanding of emulsion polymerization is that today emulsion polymerization is a mature technique and industrial formulations are based on the use of multiple monomers, multiple surfactants, and tend to be conducted under conditions that are far from the model studies that have been conducted in the academic literature. As a result, although huge strides have been made with regard to understanding emulsion polymerization, it is not really possible to predict *a priori* the full range of final properties of an emulsion polymer from its formulation. In the same way that polymer reaction engineering has benefitted hugely from an improved understanding of fundamental kinetic constants over the past 30 years, future work needs to be directed toward isolating some of the kinetic and thermodynamic parameters that would allow for a more critical analysis of the current emulsion polymerization models that are present in the literature.

### 2.2 Monitoring and Control of Emulsion Polymerization

Emulsion polymers are products-by-process whose final properties are governed by multiple characteristics. The limited fundamental knowledge discussed in the previous section makes its robust and reproducible production challenging. Therefore, there is strong academic and industrial interest in developing on-line monitoring and control strategies for emulsion polymerization reactors.^56^ However, the developments in this field have been hindered by the lack of development of on-line sensors. In fact a relatively limited number of properties can be measured directly on-line,^57,58^ although others can be estimated indirectly from on-line measurements or by using mathematical models of the polymerization process as soft sensors. Some other aspects of the polymerization process like mixing conditions are not detectable.

For properties that are readily measured, there are often control strategies that can allow for reproducible synthesis, even in the event of process disturbances. For example, copolymer composition, monomer conversion, and polymerization rate can be estimated from direct measurement of the free monomers in the reactor and can be manipulated by altering the monomer/initiator feed. Gas chromatography (GC)^59−61^ and spectroscopic techniques such as near-infrared spectroscopy (NIRS),^62,63^ nuclear magnetic resonance (NMR),^64−66^ ion mobility,^67^ and Raman spectroscopy^68−71^ have all been used for the direct on-line monitoring of the free monomer in emulsion polymerization. Among these techniques, Raman spectroscopy is the best suited spectroscopic technique for monitoring the individual free monomer concentration in an in-line process, with successful examples on monitoring^70^ and on-line control of copolymer composition reported for emulsion polymerization including its implementation in an industrial pilot plant.^71^ Reaction calorimetry has also been successfully implemented to monitor the free monomer concentration in homopolymerizations in which the heat of the reaction, which is proportional to the rate of the polymerization, is measured.^72^ In the case of copolymerization, the individual free monomers are nonobservable but can be estimated reliably using the Mayo–Lewis equation as a soft sensor. There are many successful examples of the implementation of calorimetry for on-line monitoring and control of copolymer composition^73−75^ in different emulsion copolymerization reactions including copolymerizations and terpolymerizations of monomers with different hydrophilicity in which the partitioning of the monomers between the phases was also considered.^29,76^ It should be noted that there is an intrinsic limitation in on-line monitoring of the free monomer in case of starved conditions (very low monomer concentration) and the use of functional monomers in which the error of the free monomer estimation was shown to be high.^77^

Despite ongoing research, on-line monitoring of the average particle size and particle size distribution is still a challenge. The average particle size (dp) can in principle be monitored by in situ NIR spectroscopy^78^ and Raman spectroscopy.^79^ However, despite some successful examples,^62,80−82^ the prediction of particle size by NIR spectroscopy is known to be poor due to the large difference between typical size of particles in emulsion polymerization and the wavelength of the NIR radiation. Raman spectroscopy typically gives good predictions of the average particle size, even at high solids content (up to 48%);^63^ however, for that, one needs to overcome the chemometric challenges of the measurement of dp by Raman using multivariate linear or nonlinear calibration models. In the lack of a proper on-line sensor, artificial neural network (ANN) models have been used as soft sensors to monitor the dp;^83,84^ however, these kinds of soft sensor are only valid in the region that they are trained. Photon density wave spectroscopy (PDW) is a promising dilution-free, calibration-free spectroscopy technique which has shown potential use for in-line monitoring of the reaction and particle size in emulsion polymerization.^85,86^ However, to obtain particle sizes from PDW spectroscopy, proper scattering models for complex emulsion polymerization systems are needed that take into account temperature-dependent physicochemical properties (e.g., refractive indexes of polymer and monomer) as well as measuring monomer conversion. Furthermore, the determination of the PSD by in-line PDW has not been demonstrated. It is worth highlighting that even in the case where hard sensors for on-line monitoring of particle size and PSD will be developed in the future, on-line control of these characteristics may remain a challenge. Particle nucleation and coagulations are points of no return because if they occur reversal is not possible.^87^ Early detection and diagnosis is the classical way of dealing with this type of problem.^88^ However, the sensors available are not able to detect the onset of secondary nucleation and coagulation. For coagulation, conductivity measurements can provide information about the ionic surfactant concentration which could be then related to the changes in the surface area of the polymer particle which could be linked to nucleation or coagulation phenomena.^89^ The Mathematical models are of little use here because, to the best of our knowledge, they are not accurate enough for real formulations.

One of the most important features of any polymerization process is the molar mass, which should be maintained within a relatively narrow range for quality control purposes. Unfortunately, no online sensor is available to measure average molar mass and molar mass distribution. However, successful examples have been reported in the literature based on using a combination of on-line measurements and a soft sensor to estimate these nonobservable characteristics. These cases are limited to linear polymers which were produced in the presence of chain transfer agent^90,91^ or reversible addition–fragmentation chain transfer (RAFT) emulsion polymerization using calorimetry.^92^ In the presence of the chain transfer agent, the kinetic chain length of the linear polymer can be estimated from the on-line measurement of the free monomer if the kinetic rate coefficients are known. With the use of this approach, on-line control of MWD of linear polymers has been reported.^90,93^

In general, most reports of monitoring and control have focused on single characteristics. However, the final properties of emulsion polymers are governed by multiple characteristics, and therefore, it is important to be able to monitor and control multiple different characteristics simultaneously. One option for this is through the use of the automatic continuous on-line monitoring of polymerization reaction (ACOMP) equipment,^94,95^ although this comes with the drawbacks of a continuous waste stream, potential plugging of the system, and a delay in obtaining information. In the case of emulsion polymerization, ACOMP can be used to monitor both polymer and colloidal characteristics (such as conversion, average molar mass, intrinsic viscosity, and monomer droplet and polymer particle size) on-line.^96^ Although the simultaneous on-line monitoring of certain characteristics of emulsion polymerization is possible, simultaneous on-line control of different characteristics is elusive and has only been reported in a few cases. Simultaneous control of copolymer composition and molar mass distributions of linear polymers is one of the scarce cases in which more than one characteristic of the emulsion polymer was controlled.^91,97,98^

Despite all the developments in this field, some characteristics of emulsion polymers have not yet been possible to monitor/control on-line through any technique. One of the main characteristics for which control strategies do not exist is the molar mass (and molar mass distribution) of branched and cross-linked polymers, although attempts at open-loop control of these polymers have been reported in the literature. Another unsolved area is the monitoring and control of surface composition, which is an almost totally unexplored area. Finally, in the case of particle morphology of structured polymer particles, even its off-line characterization is still an area of active research.^48,99^ Nonetheless, there have been attempts in the literature to develop robust mathematical models for describing the particle morphology^100−107^ and to use them as a soft sensor.^108,109^ However, there is still a long way to go and their use requires knowledge of a large number of system-specific parameters. Recently, there have been attempts at open-loop control^110^ and on-line control of the particle morphology based on strategies that aimed at controlling parameters affecting the particle morphology^111^ (e.g., viscosity of the matrix and process time) and control over final particle morphology by using master trajectories obtained from a given reference process as set points.^112^

Due to the lack of both hard and soft sensors, research in the upcoming years should be focused on developing new sensors for those characteristics for which on-line monitoring has not yet been possible. In the absence of robust and fast mathematical models to be used as soft sensors, machine learning^113^ may offer a potential solution as artificial intelligence methods can learn complex behaviors. The use of artificial intelligence tools/models in polymer reaction engineering and, in particular, in the area of emulsion polymerization needs to be explored. In addition, simultaneous on-line control of different characteristics should be a focus of research. In the upcoming years, advances in hard sensors are expected in the area of on-line monitoring of average particle size and PSD with the developments of new hard sensors such as OptoFluidic Force Induction OF2i technology.^114^

### 2.3 Understanding and Improving Latex-to-Film Transition

**Figure 4:**
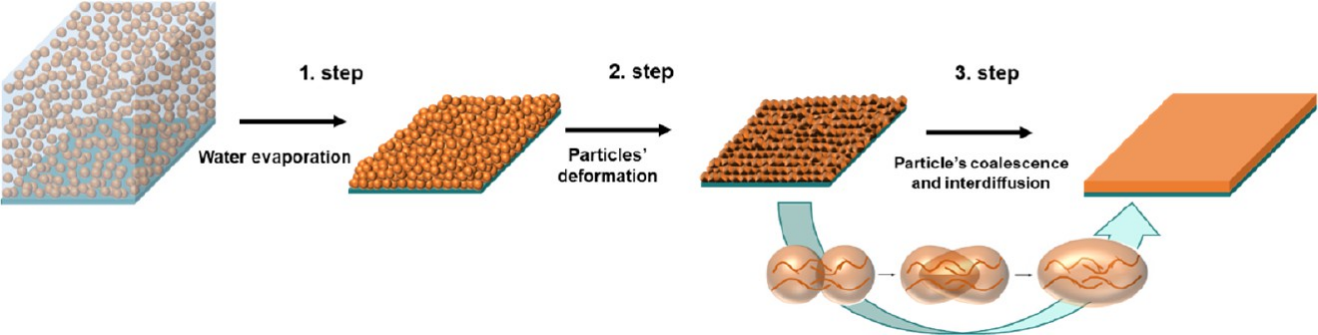
Film formation process.

So far, the discussion has been limited to the synthetic process through which polymer colloids are produced, but there are also issues in the application of colloidal polymers, even in established areas such as coatings. One particular issue that affects numerous applications is the transition between the colloidal state from which the latex is applied and the final dried polymer film, a process that is central to the widespread use of polymer colloids as binders in paint formulations. The film formation process of colloidal polymers is constituted by three main steps that are water evaporation, particle deformation, and finally coalescence and interdiffusion of polymer chains between neighboring particles (see Figure 4).^115^ As these last steps require the polymer to be in a relatively soft state, i.e., above its glass transition temperature (*T*_g_), the minimum temperature at which a film can be formed (minimum film formation temperature, MFFT) is closely related to the *T*_g_ of the polymer forming the film.

If the polymer film is to be used as an adhesive, the relation between the MFFT of the latex and the *T*_g_ of the polymer does not represent a major issue, as the adhesives usually possess a *T*_g_ well below the application temperature of the films. However, in many coating applications, it is desirable to use a polymer with a *T*_g_ higher than the temperature at which the film is formed in order to enhance mechanical performance. As such, these latexes have MFFTs higher than room temperature, which prevents the particle’s coalescence-interdifussion process (Figure 4) and hinders their application to produce hard and homogeneous films at moderate temperatures. This is a major factor in the difficulty in transitioning away from solvent-based products, where the polymers are inherently plasticized during the drying process. In water-based systems, this issue can be solved by the addition of coalescing aids to the latex before film formation. These coalescing aids, which are generally organic molecules of low molar mass, plasticize the hard polymer phase, reducing the *T*_g_ and therefore the MFFT of the latex. Once the film is produced, the coalescing aids evaporate, leaving behind a film with high modulus.^115^ However, this approach, even if effective, results in the release of organic coalescing aids to the atmosphere, thus masking the environmental benefits of waterborne coatings. As regulation over volatile organic content becomes increasingly strict, new approaches are required to obtain films with good mechanical properties with low MFFT latexes.

In light of this, several approaches have been proposed to overcome the “film formation dilemma”, with the aim of reducing the MFFT, while still obtaining hard coating films. The use of reactive coalescing aids (RCA) is one of the approaches that has appeared in the last years. RCAs act as conventional coalescing aids, lowering the MFFT of the latex, but their release is prevented by reaction with themselves or with the polymer present in the latex such that they are incorporated into the final film. While in some cases the reaction does not require any external trigger,^116,117^ the RCA usually requires the incorporation of additional initiator to the latex^118^ or the curing of the latex by UV irradiation.^119^ Even if attractive, this approach requires the use of complex organic chemistry to synthesize the RCAs which typically utilize dicyclopenteneyloxy acrylates,^118^ oxopentanoate functionalized compounds,^119^ or hydroxyethyl sulfones.^117^

Hydroplasticization has also been proposed to reduce the MFFT of latexes, by using water to plasticize hydrophilic groups present in polymer latexes.^120−123^ However, at low relative humidity, the film formation process can be significantly affected due to the higher rate of water evaporation and, therefore, the environmental conditions in which hydroplasticization is a viable strategy are limited. Furthermore, the water sensitivity of the final film can be compromised by the presence of the hydrophilic groups. Recently, the use of oligomers produced in situ by the addition of a chain transfer agent at the end of the main polymerization has been described as a scalable method to reduce the MFFT of hard latexes, without significantly affecting the mechanical properties of the final films.^124^ IR-assisted sintering of latexes containing hard latexes may also be a useful approach to produce hard films at lower energy consumptions than conventional ovens.^125,126^

Another potential way to reduce the MFFT is to use a low *T*_g_ film forming polymer in combination with a high *T*_g_ polymer phase, either in separate particles (in latex blends) or in multiphase particles. In this sense, Geurts et al. produced high performance coatings with low MFFT using blends of small, hard particles and large, soft particle sizes.^127^ For this strategy to be successful, stratification of small and big particles must be avoided in order to obtain homogeneous films. Multiphase particles containing soft and hard phases have also been synthesized. This avoids any potential stratification during film formation but comes with additional complications in the synthesis and the film formation processes. For instance, hard core/soft shell particles have been sought, in order to produce films with low MFFT reinforced by the hard cores.^128^ However, the mechanical improvements attained with this morphology are not substantial, due to the lack of connectivity between the hard phases located in the cores of the particles. Better mechanical properties can be obtained when the hard polymer is primarily located at the particle surface. In this case, the optimization of the amount of the hard phase and its morphology can lead to low MFFT latexes with highly improved mechanical properties, due to formation of a more interconnected hard phase.^129−131^

Alternative approaches have not focused on decreasing of the MFFT of the latex but rather at increasing the modulus of the polymer by cross-linking, either after or during film formation. It has to be pointed out that the cross-linking reactions must occur during or after the film formation process, because if they happen earlier, for instance during the main polymerization process, they may completely hinder the film formation.^132^ In other words, diffusion of polymer chains must be faster than cross-linking reactions to obtain hard and homogeneous films.

The primary approach to achieve cross-linked films in commercial systems is through chemical cross-linking. Several cross-linking chemistries have been tried, such as melamine-hydroxyl, aziridine-carboxylic acid, carbodiimide-caborxylic acid, oxirane or oxazoline-carboxylic acid, isocyanate-hydroxyl, or acetoacetoxy-amine.^132^ Traditionally, the cross-linking agent has been mixed with the functionalized latex before film formation, in order to trigger the cross-linking reaction during film formation. For instance, Koukiotis et al. used adipic acid dihydrazide as a cross-linker in diacetone acrylamide functionalized latexes, and the films obtained after drying at room temperature for 14 days presented higher tensile stresses and lower elongations at break, together with improved solvent resistance than non-cross-linked counterparts.^133^ However, this approach presents problems from the application point of view, as the use of many of the most common low molar mass cross-linking agents is under scrutiny due to chemical classification, labeling and packaging (CLP) and registration, evaluation, authorization, and restriction of chemicals (REACH). One way to overcome this problem is to include the reactive moieties in separate polymer particles. For example, Tariq et al. produced acetoacetoxy- and amino-functionalized latexes separately and demonstrated that the film obtained upon mixing was harder and more water resistant than the non-cross-linked latexes.^134,135^ The major issue with this strategy is that unlike the reaction between low molar mass compounds, which can be rapid even at low temperatures, when the reactive functional groups are linked to polymer chains, the rate of cross-linking is significantly reduced due to the limited diffusion of the polymer chains, and therefore, elevated temperatures may be required for curing.^132,134−136^ Another aspect to be taken into consideration when using chemical cross-linking is the stability of the latexes that contain complementary reactive moieties, which may be limited, resulting in aggregation of particles.^137,138^ This can be solved by improving the colloidal stability of the polymer particles,^139^ by the activation of reactive groups by dehydration during film formation^140^ or by producing the mixture just before the coating application, like in a two-pack system.

As an alternative to chemical cross-linking, physical interactions, driven by ionic interactions or by H-bonding, have been proposed to improve the mechanical properties of films cast from latex dispersions. For example, positively charged latexes (containing for instance 2-(dimethylamino)ethyl methacrylate) and negatively charged latexes (containing acrylic acid^141^ or sodium styrenesulfonate^142^) can be synthesized and mixed prior to film formation. Films prepared with blends of this type display slightly improved mechanical properties compared to the individual latexes, indicating some ionic interactions in the final film. However, one important drawback of these films is their poor water resistance, due to the increased amount of charges present in the final film. Furthermore, Argaiz et al. reported that the interdiffusion of polymer chains between neighboring particles was almost completely hindered by the presence of the ionic interactions on the surface of the particles.^142^ H-bonding physical interactions have also been reported as a way to reinforce the final films formed at low temperature.^143,144^ For example, Chen et al. synthesized ureidopyrimidone-functionalized latexes to obtain a quadruple H-bonding between polymer particles, which led to films with improved resistance to solvent.

This nonexhaustive list of approaches to solve the “film formation dilemma” should make it clear that at present there is no silver bullet to solve this problem. While many of the strategies show great promise, and indeed many are already in use commercially, it is clear that further refinements need to be made to the present strategies such that low MFFT, high mechanical strength films can be generated from colloidal polymers. This is particularly important as moving forward regulations are likely to become stricter, and therefore, aqueous polymer dispersions will be required for use in even more demanding applications that have traditionally been dominated by solvent-based products.

## 3 Toward the Improvement of the Sustainability and Degradability of Waterborne Dispersions

As emulsion polymerization is now a mature technology, there have been many advances that, while mainly targeting economic benefits, have improved drastically the carbon footprint of the process. For example, emulsion polymerization processes are typically conducted at high solids content (50–60 wt %), but there have been significant developments in processes involving multimodal particles size distributions that allow for the production of latexes above the theoretical limit for monodisperse particles (i.e., >70 wt %).^54,145^ This can substantially reduce transport costs and leads to significant energy savings. More recently this work has been extended through the development of “switchable surfactants” which allow for the synthesis of redispersible latexes that can in principle be transported without any additional water and then redispersed for application.^146,147^ While these efforts to improve the process efficiency and sustainability continue, there is now increasing consumer demand for products that have a significant biobased component and that are (bio)degradable. As highlighted in this section, this transition will only be possible through fundamental investigation into new monomer/polymer systems.

### 3.1 Biobased Lower Carbon Footprint Latexes

In common with many polymers, almost all emulsion polymers are currently synthesized from monomers derived from the petrochemical industry. Due to the increasing environmental awareness, together with stricter regulations with regard to greenhouse gas emissions (particularly CO_2_), there is growing pressure on industry to find biobased alternatives for its raw materials. Biobased materials are those derived from, in whole or in part, biological products obtained from biomass. Although significant effort has been directed to find new biobased alternatives (the number of publications on this topic has increased 200% in the last 10 years), the market share of biobased products in coatings is estimated to be limited to around 5%.^148^ This figure is even worse if the total production of polymers in the world is taken into consideration, where biobased polymers represent around 1% of the total production.^149^ It is particularly significant that this figure is not only low but also is not increasing at any appreciable rate and suggests that real change will only be made through fundamental research and the development of new monomer systems.

**Figure 5:**
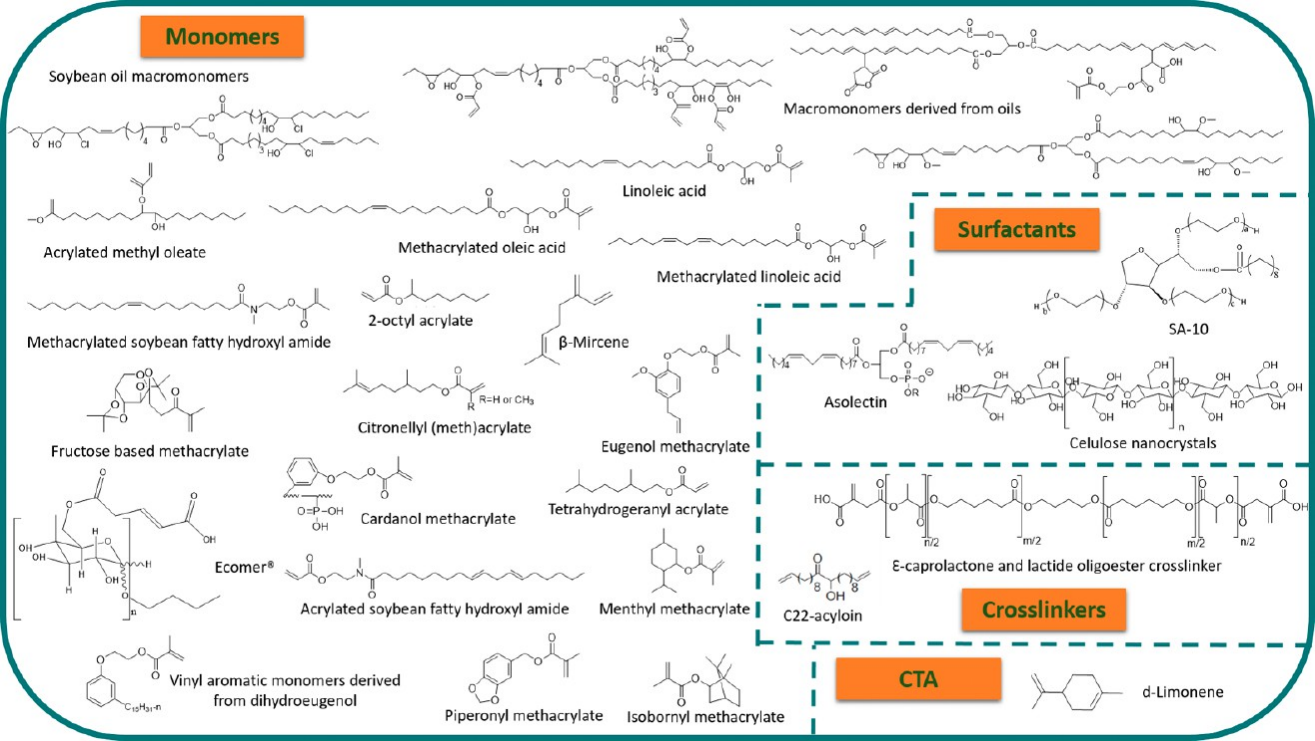
Biobased components for emulsion polymerization formulation: monomers, surfactants, cross-linkers, and CTA.

Research into biobased monomer systems has sought to cover a wide range of *T*_g_’s to be able to be applied to the various markets of emulsion polymers. An additional challenge that is present in emulsion polymers is that the monomers must have some limited solubility in water in order to ensure that the polymerization rate is not hindered by mass transport effects.^150^ In the open literature, there are numerous reviews that detail the synthesis of monomers from various biobased reactants.^151−155^ The renewable starting materials can be divided in five main families: carbohydrates, vegetable oils, lignin, terpenes and proteins, and amino acids.^156^ In the case of (mini)emulsion polymerization, monomers obtained mainly from carbohydrates (fructose^157^ or dextrose^158,159^) and from vegetable oils (soybean oil,^160−165^ sunflower,^166,167^ olive oil,^168^ rapeseed oil,^169^ plant oil,^170^ castor oil,^171−173^ and clove oil^174,175^) have been used. Terpene-based monomers have also been incorporated using emulsion polymerization^176−179^ and fully terpenoid-based acrylic PSAs with a high biobased content have also been reported.^180^ Some of these monomers are summarized in Figure 5.

With all this work in mind, it seems that there already exists a wide portfolio of starting materials to produce either coatings or adhesives by emulsion polymerization. The major issue in practical implementation is the availability of the raw materials in sufficient quantity and quality for significant replacement of conventional petrochemical-based monomers. It should be also considered that the composition of the natural feedstocks may vary depending on the region that the plants are grown, which makes the commercial production of biobased monomers even more complex. In the absence of 100% biobased monomers, a viable alternative is the partial substitution of petrochemical-based monomers or use of monomers with high biobased content as recently reported by Badía et al.^181^ In any case, to achieve 100% biobased products, it is not only the monomers that must be considered; the rest of the polymerization reagents (surfactant, cross-linkers, CTA, ...) (see Figure 5) and the ingredients of the coatings or adhesive formulations must also be biobased.^182^ Currently there are few works in which different reactants of the formulation are biobased, and therefore, future work should be directed toward using biobased components in all aspects of the formulation.

Apart from obtaining a biobased product, from the sustainability point of view, the carbon footprint of these materials should also be considered. In many cases, the synthesis of the biobased monomers is long and low yields are achieved and, hence, not only does the process become more expensive but also the final carbon emissions of the material are higher. This means that even though high biobased content materials are synthesized, they may in fact be less sustainable than their petroleum-based counterparts. Moreover, if the procedures are more expensive, their price may not be competitive in the market. In this vein, Anastas et al.^183−185^ published “12 Principles for Green Chemistry”, focused on minimizing waste, using renewable feedstocks, using safe and environmentally benign substances, and atom economy to serve as a guide to minimize or eliminate the environmental impact of chemical products and processes. Dubé and co-workers have applied these principles to polymer production processes^186^ and more precisely to emulsion polymerization,^187^ citing in particular the need to use renewable feedstocks.

Going forward, the use of biobased components not only poses problems during the synthesis of the latex but also in its formulation in the final product. For example, when the main monomer of the formulation is changed, although many of the properties might be the same, others may vary, and thus, the products will have to be reformulated.^188^ This is the main reason why formulator industries are reluctant to change the raw materials in latex production. As a result, in the short term, the easiest way to increase the biosourced fraction in latexes will be through the synthesis of traditional monomers starting from biosourced raw materials (i.e., biobased methyl methacrylate).

### 3.2 Degradable Film Forming Latexes

In line with the discussion to improve the sustainability of the feedstocks of emulsion polymers, there is also a need to look at the end-of-life management of products made using colloidal polymers. One way to improve the sustainability of polymers is to develop polymeric materials that would (bio)degrade after their use; in other words, to tune the life cycle of the polymeric materials by triggering the degradation of the material after their use. The production of (bio)degradable plastics is expected to increase to 1.8 million tons in 2025.^149^ However, while some step growth polymers (e.g, PHB, PET^189^) have shown this capability, the production of fully degradable polymers (with acceptable application properties) in dispersed media (as latex) remains elusive.

In the case of waterborne systems, there are two particular challenges when targeting degradable products. The first is that latexes are produced and stored in aqueous media. This presents some obvious challenges when hydrolytically unstable components are incorporated into the polymer. The second is related to the specific uses of latexes, which tend to be applied for (semi)permanent use, where degradation is not desired. However, degradable polymer films may find application in single use applications, as paper coatings for food packaging or as removable adhesives. For instance, one of the major problems to obtain good quality glass during the reusing of glass bottles is the presence of the adhesive labels. Currently, the adhesive labels are removed immersing the bottle into a basic aqueous solution at high temperatures.^190^ If the adhesive contains cross-linkers with ester groups (e.g., allyl methacrylate), the adhesive would be hydrolytically degradable. This offers a way for the glass industry to increase the quality of the glassware in a sustainable way. However, introducing aliphatic ester groups (lactic acid and caprolactone) in the cross-linker of the adhesive will lead to products with faster degradation kinetics in milder conditions, reducing significantly the cost during the cleaning process of glass bottles.^191^

**Figure 6:**
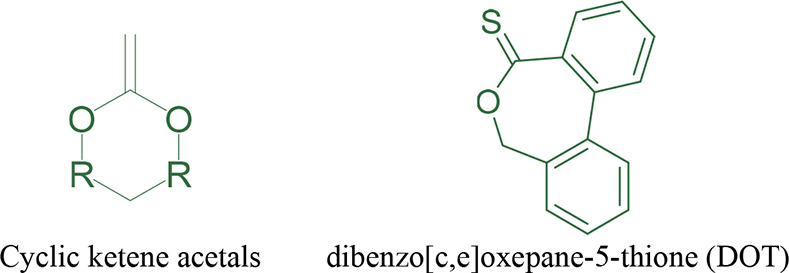
Chemical structures of cyclic ketene acetals where the R represents different alkyl groups and DOT.

**Figure 7:**
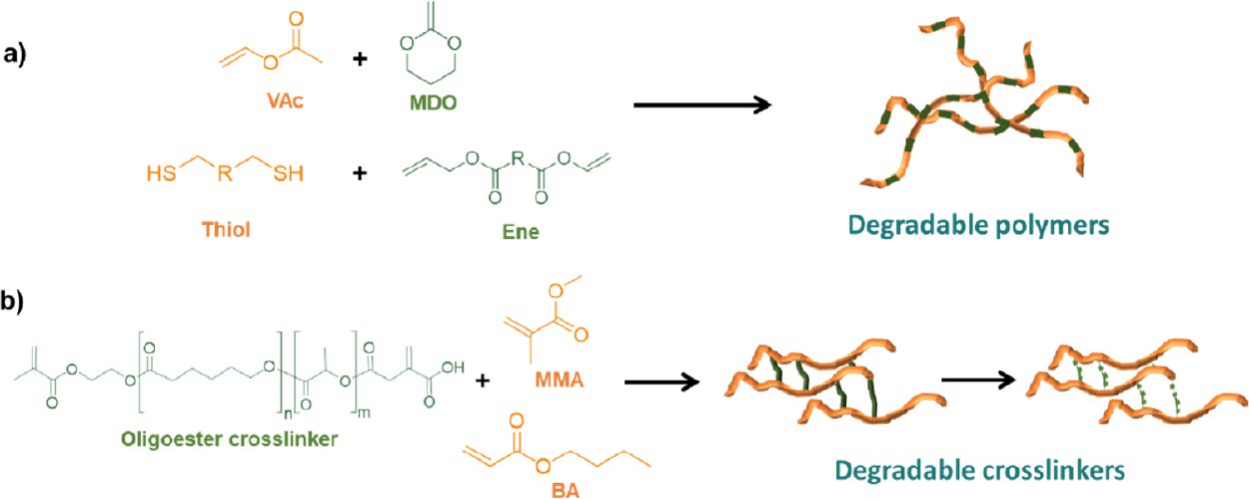
Two different approaches to introduce degradability into waterborne polymeric dispersions. (a) Degradable polymers synthesized by radical ring-opening of ketene acetals (MDO) or by thiol–ene polymerization. (b) Introduction of degradability in the cross-linking point of the polymeric network.

There are different approaches that have been proposed to introduce degradability into the polymer backbone in waterborne systems, the most common being the use of monomers containing degradable groups (see Figure 7a). For this purpose, aliphatic polyesters are good candidates due to the presence of a hydrolytically degradable ester unit in their structure. One way to incorporate ester groups is through copolymerization with cyclic ketene acetals, for example 2-methylene-1,3-dioxepane (MDO), which have a vinyl group that can undergo radical ring-opening polymerization to yield an ester in the polymer backbone (see Figure 6).^192,193^ In the open literature the ability of MDO to undergo radical ring-opening radical polymerization is well reported,^194−198^ both in free radical systems^199−201^ and in controlled/living processes.^197,202−204^ However, when copolymerizing with common monomers used in emulsion polymer such as styrene, vinyl acetate (VAc), or methyl methacrylate (MMA), the reactivity ratios of the different monomer couples can make it challenging to obtain homogeneous copolymers, especially when the homopolymerization reaction is much slower than with the other monomer.^205−208^ This may not be a major problem since incorporating only a few degradable ester units along the polymeric chain would be enough, but it does complicate the polymerization process.

Very recently, Lena et al. studied the different side reactions that MDO can undergo when copolymerizing with acrylates in solution polymerization.^209^ Moreover, reactivity ratios with different acrylates were estimated, and the optimal addition profiles for semibatch processes were calculated. This strategy allowed for reaching higher MDO conversion and better incorporation of the degradable monomer.

Although polymerization of cyclic ketene acetals in solution is now well-known, their polymerization in the aqueous phase has not been extensively reported due to the hydrolysis that the monomers can suffer in the presence of water.^207^ Carter et al.^210^ published work in which MDO was copolymerized with VAc by emulsion polymerization. Good control of the pH was key to avoid hydrolysis of the MDO in acidic conditions and, on the other side, hindered the hydrolysis of VAc which occurs under very basic conditions. However, in a more recent paper, Kordes et al. evaluated the suitability of the incorporation of MDO in homogeneous and heterogeneous systems by studying the rate of hydrolysis under different conditions. They claimed that even at optimum pH and temperature conditions, only small amounts of MDO were incorporated into the copolymer, with the majority suffering hydrolysis during the emulsion polymerization process.^211^

The polymerization of another monomer from the family of the cyclic ketene acetals, 5,6-benzo-2-methylene-1,3-dioxepane (BMDO), with MMA and styrene in miniemulsion polymerization has also been carried out.^212^ Degradability and biodegradability was proven, showing hydrolysis at basic pHs and enzymes in all the latexes synthesized, and confirming cell viability when nonionic surfactants were used. In addition, different polymers with different BMDO content have been incorporated into PMMA by emulsion polymerization. After degradation in basic pH a 90% decrease in the molar mass was observed.^213^

Another approach to obtain degradable polymers containing ester functionalities is the one proposed by Wenzel et al.^191^ They synthesized a hydrolytically degradable pressure sensitive adhesive (PSA) using a typical acrylate formulation and semibatch emulsion polymerization process but including a degradable oligoester cross-linker, made out of lactide (LA) and caprolactone (CL), in the formulation which could be used to aid recycling (Figure 7b). The properties of PSA’s were strongly influenced by the microstructure of the polymer, and thus, by degrading the oligoester cross-linker hydrolytically at basic pH during 30 min, it was demonstrated that the adhesive properties were completely lost. These PSAs could be potentially used as removable adhesives in glass bottles.^190^ The degradation products obtained through the hydrolysis of the ester groups are small alcohol and acid molecules which can be then easily removed as side products.^214^

Thiono-lactone groups have also been proposed as possible candidates to produce degradable polymers.^215^ Galanopoulo et al.^216^ recently reported the incorporation of thioester groups into emulsion polymers by the emulsion copolymerization of dibenzo[c,e]oxepane-5-thione (DOT) with *n*-butyl acrylate (BA), styrene (S), and a combination of both. DOT undergoes a similar radical ring-opening polymerization to the structurally similar cyclic ketene acetals but is more stable to hydrolysis and has better copolymerization behavior with common monomers. They were able to incorporate the DOT in a quantitative way and demonstrated that only a few units of it were necessary to obtain degradable copolymers. The same monomer has also been reported for use in systems that undergo polymerization-induced self-assembly to yield latex particles.^217^

The previous examples represent attempts to enhance the degradability of emulsion polymers produced by radical chain growth polymerization. As an alternative, thiol–ene polymerization has been proposed as a fundamentally different way to incorporate degradable groups into the polymer backbone in colloidal systems (Figure 7a). The reaction occurs between thiol and ene functional groups, and the polymerization mechanism follows a step-growth mechanism through free radical reactions. It offers some advantages such as mild conditions to carry out polymerizations with high yields and the possibility to polymerize a wide range of bifunctional monomers, which may come from renewable resources.^218−220^ However, side reactions such as homopolymerization of the ene groups and cyclization must be avoided in order to maintain the stoichiometric ratios of the functional groups.^221^ Therefore, many authors preferred to use miniemulsion polymerization with thiol–ene chemistry such that the concentration of reactants in the polymerization loci is well controlled.^222,223^ For example, using an α,ω-diene diester derived from vegetable oil, 1,3-propylene diundeca-10-polenoate (Pd10e), together with 1,4-butanedithiol (Bu(SH)_2_) has shown that latexes can be produced by miniemulsion polymerization that demonstrate degradability in both enzymatic or acidic conditions.^224^ The use of miniemulsion polymerization requires a high-energy emulsification step, which is difficult to perform at scale, and therefore, work has also been directed at producing polymer particles by thiol–ene polymerization in emulsion.^225^ Durham et al.^226^ used difunctional, trifunctional, and tetrafunctional dienes and dithiols to obtain cross-linked structures, but the molar masses were low (around 3–5 kDa). More recently, Quoc Le et al.^227,228^ have demonstrated that photopolymerization of thiol–ene systems can be used to produce latexes with solids content up to 40 wt % and reasonable molar masses (>13 kDa) by emulsion polymerization.

## 4 Novel and Emerging Opportunities for Emulsion Polymer and Polymer Colloids

So far we have been concerned with high-volume applications of polymer colloids and issues that remain in commercial synthesis and use. As mentioned in the introduction, colloidal polymers lend themselves to a wide range of applications, and therefore, in this section we will highlight some of the emerging synthetic techniques and potential applications for future research.

### 4.1 Reversible Deactivation Radical Polymerization (RDRP) in Emulsion

Reversible deactivation radical polymerization (RDRP) is now a well-established technique for the synthesis of polymers with relatively narrow molar mass distributions and controlled macromolecular architectures.^229^ Conducting these polymerizations in dispersed media can offer significant advantages over the simpler solution polymerization processes due to the compartmentalized nature of the polymerization.^230^ For example, in RDRPs that work based on the persistent radical effect [i.e., atom transfer radical polymerization (ATRP), nitroxide mediated polymerization (NMP)], physical confinement of the deactivator and the radical species in the same particle can lead to higher rates of recombination, thus enhancing livingness, although this generally comes at the cost of a reduced rate of polymerization.^231−234^ In degenerative transfer type mechanisms [i.e., reversible addition–fragmentation chain transfer (RAFT) polymerization], the compartmentalized nature of emulsion polymerization can allow for reduced rates of termination, thus allowing simultaneously for both improved livingness and higher rates of polymerization.^235−237^

**Figure 8:**
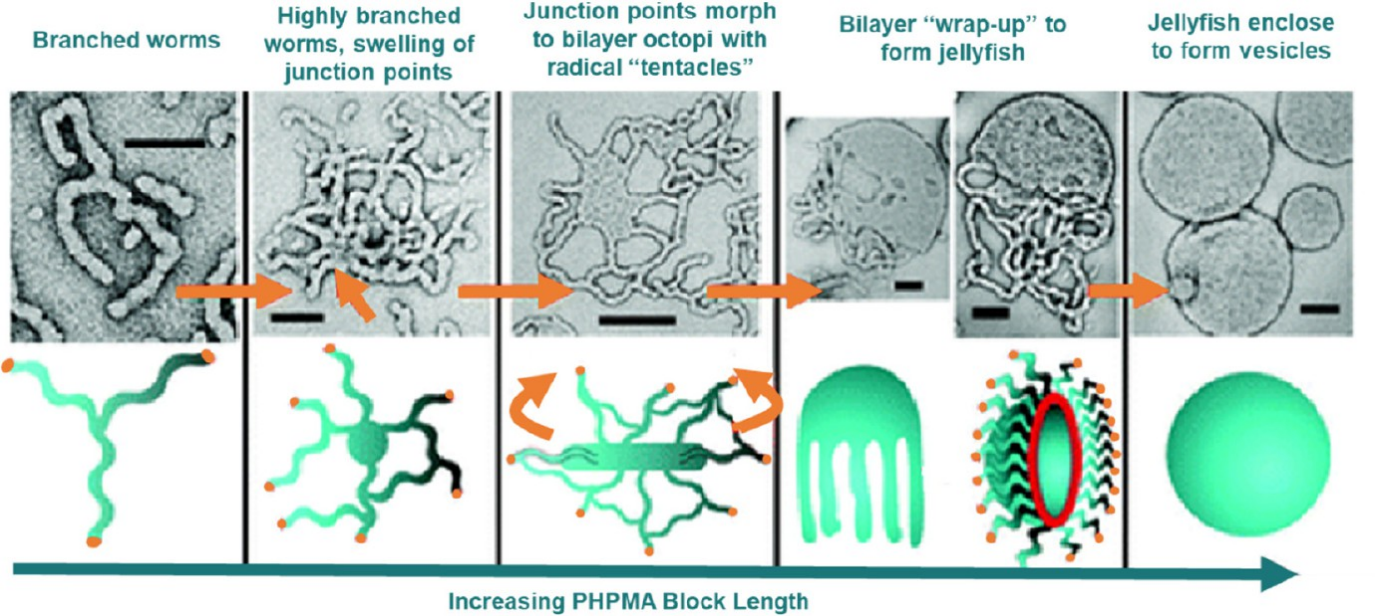
Suggested mechanism for the polymerization-induced worm-to-vesicle transformation during the synthesis by RAFT aqueous dispersion polymerization. Reprinted from ref (253). Copyright 2011 American Chemical Society.

RDRP can also be used to generate colloids that are not accessible by conventional polymerization techniques, which may open up new commercial opportunities for emulsion polymers. For example, emulsion polymerization almost invariably leads to spherical particles as a result of the minimization of interfacial energy. Although there are some strategies available to synthesize relatively simple alternatives, highly shaped anisotropic colloids are extremely challenging to synthesize (Figure 8). RDRP offers a route to such structures through the use of polymerization-induced self-assembly (PISA) in which an end-capped hydrophilic polymer is chain-extended with a second hydrophobic block to generate an amphiphilic block copolymer.^238^ The self-assembly of these block copolymers leads to colloidal structures with the morphology dictated by the nature of the block copolymer.^239,240^ Through this technique spherical micelles, cylindrical micelles, and vesicles, as well as more complex intermediate morphologies, can be readily obtained.^241,242^ The high aspect ratio of these systems and the ability to undergo dynamic morphological changes have made them of interest for a number of applications such as viscosity modifiers,^243,244^ gels for biomedical applications,^240,245^ opacity modifiers,^246^ in catalysis,^247^ and in the generation of nanostructured films.^248−251^ The major stumbling in practical use of PISA is that most reports to date have depended on the use of RAFT polymerization, which is proven to be challenging (and expensive) to scale-up. However, recent reports suggest that the fundamental work performed with RAFT-based PISA can be extended to more scalable systems^252^ by using addition–fragmentation chain transfer-type polymerizations, which avoid many of the drawbacks of RAFT at the expense of reduced control over the polymerization.

### 4.2 Waterborne Latexes Containing Covalent Adaptable Network

Covalently cross-linked polymer systems, i.e., thermosets, are used in numerous applications due to their excellent mechanical and thermal integrity. However, the presence of the cross-linked structure makes the shape and nature of the polymer permanent and largely intractable. The desirable combination of a covalently cross-linked polymer structure with the capability for a triggerable reversion of the cross-linked polymer structure has recently been coined a covalent adaptable network (CAN). Bond exchange typically proceeds by one of two mechanisms: either a “dissociative” process in which cross-links are cleaved into their individual constituent reactive partners before regenerating or an “associative” process in which a pendent reactive group within the network undergoes a substitution reaction with an existing cross-link.^254^

In the field of latexes, the incorporation of dynamic bonds has been much less explored than in bulk materials. Latexes provide certain advantages in comparison to bulk materials as stated in the above sections, in terms of processability, heat removability, and scalability. In 2018, Montarnal and co-workers presented for the first time the synthesis of vitrimer nanoparticles using incompatible epoxy–acid vitrimer precursors by miniemulsion polymerization.^255^ They found that the choice of surfactant was critical to maintain the stability of the emulsion, especially when vitrimer curing was carried out at high temperatures, and that conventional surfactants such as SDS had to be used in large amounts. They managed to mitigate this by the generation of carboxylate surfactants in situ from the dimer fatty acid, which greatly accelerated the epoxy-acid addition and also improved the malleability of the resulting vitrimer films.^256^ The authors found that this chemistry could be integrated with bioinspired waterborne nanocomposites based on cellulose nanofibrils and demonstrated that by increasing the vitrimer content, the overall ductility increases, whereas the stiffness is regained through activation of the cross-linking and transesterification reactions.^257^ These approaches to dynamic chemistry may have application in solving the “film formation dilemma” discussed in section 2.3 by allowing for cross-linked materials that are still capable of some degree of reorganization.

Besides acrylates and epoxy waterborne systems, polyurethane dispersions containing dynamic bonds have been also investigated in the literature.^258^ Due to the inherent incompatibility of isocyanate groups with water, polyurethane-based dispersions are almost exclusively prepared by first generating a polyurethane containing a stabilizing diol most commonly dimethylolpropionic acid (DMPA) and dispersing this in water after the polyurethane is neutralized with a base, commonly triethylamine (TEA). Irusta et al. exploited this chemistry to incorporate different chain extenders to provide dynamic behavior. They managed to incorporate not only aromatic disulfides for autonomous healing but also coumarin-based diols for UV-light responsive materials.^259^ These disulfide-containing polyurethane dispersions have been shown to generate film forming materials that are capable of self-healing under certain conditions and may prove useful in certain coatings applications.^260,261^ Due to versatility in the chemistry, some supramolecular interactions have been also introduced in waterborne polyurethane dispersions to provide some dynamic character and to improve mechanical strength.^258^

As suggested by Montarnal and co-workers, in principle, the preparation of latex vitrimers could be extended to other covalent adaptable networks. However, care must be taken as some of these exchange reactions may be sensitive to water. It is clear that given the large volume of latex production, chemistries that are easily scalable should be implemented. One potential alternative for preparing CAN latexes in a straightforward and scalable method is to use the strategy recently reported by Sumerlin and co-workers for the generation of vinylogous urethane vitrimers using conventional radical polymerization.^85,262^ The copolymerization of the commercially available and inexpensive monomers styrene and (2-acetoacetoxy)ethyl methacrylate produced β-ketoester-functional network precursors on a multigram scale, which could be cross-linked with diamines to yield thermally robust vitrimer materials. Chemistries such as this one or others that could be easily transferable to emulsion polymerization should be investigated for the preparation of covalent adaptable network nanoparticle latexes.

### 4.3 Waterborne Systems for CO_2_ Adsorption

The continuous increase of atmospheric CO_2_ and its negative climate impact have led to a large search for novel strategies for CO_2_ removal for which colloidal polymers may be useful due to their high specific surface area. One of the main approaches developed is the capture and sequestration of CO_2_ using solid adsorbents. A critical issue to achieve high performance by this approach is the development of adsorbents that are produced with environmentally friendly methods at low cost and present high performance in CO_2_ adsorption and separation from other gases (N_2_ or CH_4_). The requirements for high performance adsorbents are different, depending on the conditions in which they will be used. Nevertheless, as generalized by Oschatz and Antonietti in a recent review article,^263^ in addition to highly selective capture capacity in the gas mixture, it is of high importance to have adsorbents with fast adsorption/desorption kinetics that can be regenerated under mild conditions (either by pressure- or temperature swings) and that present high resistance against impurities and moisture. The physicochemical properties of emulsion polymers, the environmentally friendly way of their production, and the easy tuning of the surface functionalities, along with high hydrophobicity that ensures stable function in humid atmosphere, place them as one of the most attractive building blocks for the synthesis of high performance solid CO_2_ adsorbents. Usually the colloidal polymer particles have been post-treated to either produce porous polymeric structures,^264,265^ postfunctionalized to be used in fluidized bed reactors,^266,267^ or have been included into porous matrix of other materials to produce composite adsorbents.^268,269^

Inspired by the commercial liquid amine CO_2_ absorption process, which until very recently was the only commercialized technology for CO_2_ capture and sequestration, nitrogen-containing moieties have been placed within the main polymer chain or added by postproduction functionalization of the emulsion particles. Morbidelli’s group^264^ synthesized polyacrylonitrile (PAN) particles by emulsion polymerization, which were converted into porous materials by controlled destabilization of the original particle dispersion, followed by drying and grinding. Moreover, the porous particles were subjected to thermal treatment to develop microporosity, resulting in a high BET (Brunauer, Emmett, and Teller) specific surface area of about 400 m^2^ g^–1^ and CO_2_ adsorption capacity of about 3.56 mmol g^–1^ (at 273 K and 1 atm). The authors stated that the capture capacity was mainly affected by the presence of micropores with a diameter smaller than <0.7 nm and that the acid/base character of the nitrogen-bearing polymer chains did not affect the final uptake. On the other hand, Shi et al.^265^ used a much simpler and more scalable layer-by-layer process based on polymer colloids to produce hyper-cross-linked polymer films with tunable thickness, composition, and pore size. The polymer colloids based on poly(styrene-*co*-butyl acrylate) were produced by emulsion polymerization and were postfunctionalized with −NH_2_ moieties using anhydrous ethylenediamine. These particles were deposited by a dipping technique, resulting in porous films with BET specific surface area as high as 605 m^2^ g^–1^, high CO_2_ adsorption capacity of 12 mmol g^–1^ at 273 K and 1 atm, and excellent stability in cycle operations (demonstrated in 6 cycles).

Although colloidal polymers have relatively large surface area, the surface area can be enhanced through the generation of highly porous structures within the particles. For example, Nabavi et al.^267^ used emulsion polymerization to produce spherical cross-linked particles made of acrylamide and ethylene glycol dimethacrylate. Using small amounts of porogenic solvents (acetonitrile and toluene) during the synthesis, mesoporosity was developed within the particles. The particles were molecularly imprinted by using oxalic acid as a template, which has a spatial structure very similar to two CO_2_ molecules with their C atoms sitting back-to-back and O atoms pointing in opposite directions, resulting in nanocavities decorated with amide functionalities. The BET surface of these particles was quite high (up to 457 m^2^ g^–1^), and they presented relatively high CO_2_ capture capacity at low pressure conditions (0.62 mmol g^–1^ at 298 K and 0.15 bar). Kaliva et al.^266^ reported the synthesis of highly cross-linked poly(styrene-*co*-divinylbenzene) particles by emulsion polymerization. The authors stated that the rigid and controlled microstructure of the polymer chains hinders their efficient packing that might give rise to high porosity. Rather modest BET specific surface area was achieved (up to 205 m^2^/g) with 0.9 mmol g^–1^ CO_2_ absorption capacity (at 268 K and 1 atm) and according to the authors high performance for a CO_2_/CH_4_ gas separation (selectivity up to 12, determined by IAST).

**Figure 9:**
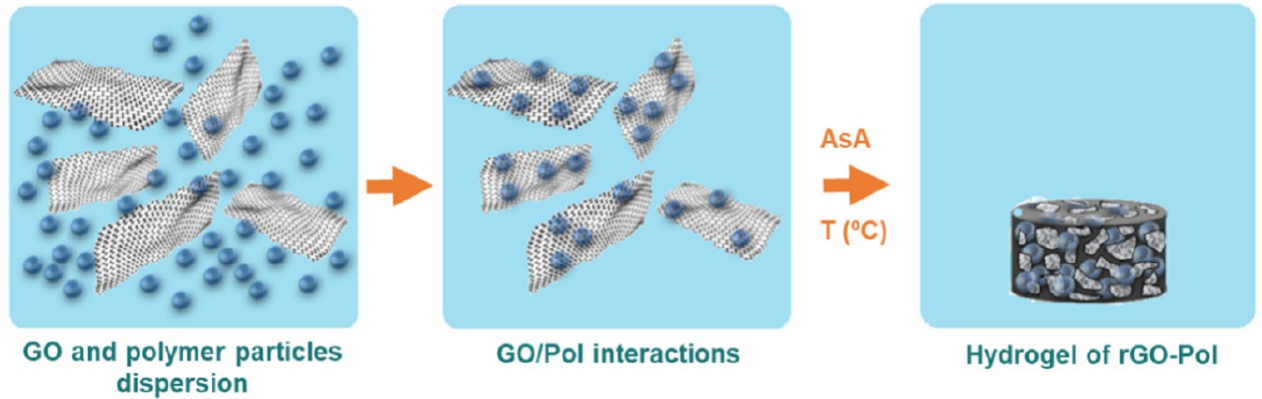
Schematic representation of the preparation of the monolithic composites made of reduced graphene oxide/polymer in aqueous dispersion of graphene oxide nanoplatelets and (gray) and polymer nanoparticles (blue). Reprinted with permission from ref (269). Open Access.

A different approach reported by Politakos et al.^268,269^ is the generation of three-dimensional (3D) monolithic composite materials based on reduced graphene oxide (rGO) and functional polymer particles (see Figure 9). They reported a low-energy synthetic process based on GO reduction-induced self-assembly of rGO platelets decorated with polymer particles (Figure 9), synthesized by emulsion polymerization and functionalized in situ during the polymerization reaction. It was shown that the reduction parameters, temperature, and reducing agent (ascorbic acid) quantity affected the morphology, functionalization, and textural properties, including the BET surface area, giving rise to a range of various porous monoliths made of either neat rGO or rGO/functionalized polymers, with the highest BET surface area of 328 m^2^ g^–1^ and 235 m^2^ g^–1^, respectively. Addition of epoxy-functionalized particles to the rGO monolithic structures decreased the BET surface area, but the CO_2_ adsorption was doubled with respect to the neat structures to almost 4 mmol g^–1^ of solid sorbent (at 298 K and 1 atm). The observed phenomenon of increasing CO_2_ adsorption even at lower specific surface area indicates that there were two competing effects. On the one hand, the polymer particles present on the graphene surface affected the self-assembly process, acting as spacers between the individual platelets, preventing closer platelets stacking and formation of micropores (resulting in lower surface areas). On the other hand, the polymer particles increased the functionalization, thus offering more adsorption sites for CO_2_ molecules. These results demonstrated that in the composite structures, the CO_2_ selective CO_2_ adsorption performance is an interplay between the materials’ textural properties and the level of functionalization.

In the recent work of the same authors, as a strategy to increase the BET surface area, Barbarin et al.^270^ reported the synthesis of cross-linked particles by emulsion polymerization based on MMA and divinylbenzene (DVB), which afterward were incorporated into the 3D graphene monolithic structures following the scheme in Figure 9. The presence of the cross-linked particles in the monoliths contributed to develop further micro- and mesopores, increasing the BET surface area and CO_2_ adsorption capacity up to 100% with respect to graphene monoliths containing non-cross-linked MMA polymer particles. Moreover this strategy permits incorporation of a higher quantity of polymer into the composite structure (40 wt %), which might be a way to improve the durability and stability in the cycle operation and to decrease the costs of the materials.

Even though there is a limited number of reported works, it has clearly been demonstrated that colloidal polymer particles are useful building blocks for the design of different porous adsorbents. This is due to the easiness of the synthesis procedure (emulsion polymerization) that offers high capacity production of polymer particles in aqueous media with the simultaneous opportunity to functionalize them with limitless functionalities.

### 4.4 Energy Applications

**Figure 10:**
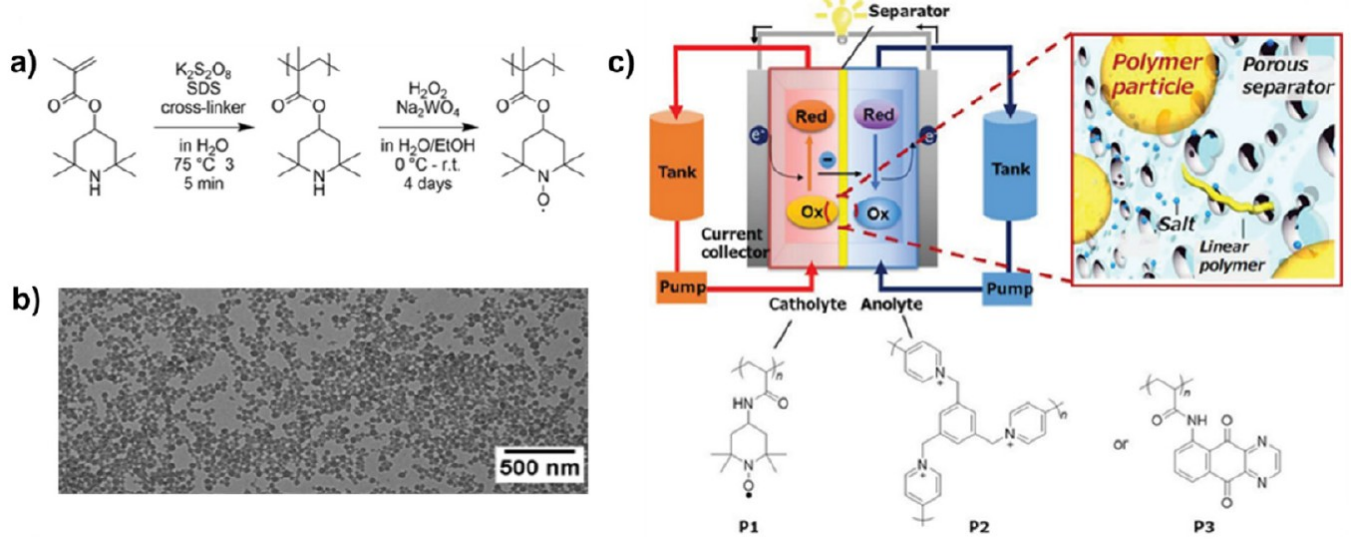
(a) Synthesis of PTMA -based polymer by emulsion polymerization followed by deprotection. (b) SEM image of emulsion polymer resulting from this process. (c) Redox flow battery based on the use of TEMPO, viologen, or diazaanthraquinone-functionalized colloidal polymers. Reprinted with permission from ref (276). Open access. Reprinted from ref (280). Copyright 2019 American Chemical Society.

One application that has recently become targeted is the use of redox active nanoparticles as cathode materials, where, similar to the CO_2_ sorbents discussed above, the high surface area of colloidal systems lends itself to good performance. For these types of applications, the latex needs to contain redox active groups.^271,272^ Since the redox active components tend to influence the free-radical polymerization process,^273^ in most cases, the polymers are synthesized with the monomers in some deactivated form (by protection of the active group) and are subsequently converted to the redox active state. For example, Pirnat et al. reported the emulsion polymerization of dimethoxy styrene-based polymers with small amounts of cross-linker and subsequently deprotected the polymer to give catechol-containing polymer colloids.^274^ The redox potential of such polymers can be adjusted by the inclusion of vinylpyridine, which acts as a proton trap in organic electrolytes.^275^ Another popular choice for emulsion polymers targeted at cathodic materials is the use of nitroxide-containing polymers. For example, Muench et al. have recently reported the synthesis of poly(2,2,6,6-tetramethyl-4-piperinidyl-N-oxyl methacrylate) (PTMA)-based electrodes by emulsion polymerization of a piperidyl methacrylate and subsequent oxidation to a nitroxide (Figure 10).^276^ In that case, the particle size was observed to play an important role, with smaller particles demonstrating lower capacity losses.

Redox active colloidal polymers have also been proposed for use in redox flow batteries.^277−279^ Organic redox flow batteries based on polymer solutions are well-known but tend to suffer due to the limited solubility of the polymers in the electrolyte and the relatively high viscosity of polymer solutions, both of which can be overcome by using colloidal dispersions. For example, a dispersion polymerization process was used by Hatakeyama-Sato et al. to generate a hydrophilic colloidal dispersion of polymers containing a nitroxide (see Figure 10).^280^ The use of colloidal particles allowed them to exceed the solubility limit of the corresponding polymers and also prevented any crossover between the two components of the battery.

### 4.5 Biomedical Applications for Latexes

Polymeric particles with nanometer and micrometer dimensions are particularly interesting for the biomedical field.^281^ Emulsion, microemulsion, and miniemulsion polymerizations have been shown to be very promising and with flexible synthetic techniques, with pros and cons when it comes down to the biomedical use of their products. In the application arena, latexes have found uses across a broad spectrum of systems, where typical examples involve their use in the transport of active pharmaceutical ingredients (APIs), as platforms for the development of diagnostic techniques, and for tissue engineering.

Key features that are crucial to optimize such kinds of applications involve the size of the particles, their size distribution, their shape and surface properties (electrostatic charge, chemical functionalities, etc.), the purity of the products, and the production scale. Size, size distribution, and shape are crucial in order to control the reproducibility of the pharmacological behavior of the particles.^282^ Surface properties also need to be precisely controlled, as they determine the type of biointerface that is formed once the particles get in contact with biological matter such as blood, cells, and tissues.^283,284^ As examples, surface functionalization with peptides like arginylglycylaspartic acid (RGD) has enabled the attachment of the particles on cells;^285^ surface charge is typically used to modulate the biocompatibility of the nanomaterials and their uptake by cells and their intracellular fate, whether this involves targeting a certain organelle or the release of the cargo at the certain time and location inside or outside the cell.^286^

The purity grade of the products is particularly important when the impurities may have some pharmacological activity. For particles prepared by emulsion techniques, being able to remove the surfactants from the products is of high importance, as often surfactants are able to disrupt cell membranes, open gaps in tissues such as the skin, and have low biocompatibility. Moreover, scalability of the particle production in clinical grade should also be considered.

A huge variety of nanoparticles have been prepared using emulsion techniques for drug delivery. The most common strategy involves the cross-linking of monomers or functionalized polymers that are templated in a form of (nano)droplets in a emulsion process. This method typically results in particles with a rather hydrophobic interior and a hydrophilic shell, the former being ideal for the encapsulation of hydrophobic drugs and the latter ideal to minimize undesired surface interactions. A seminal example is that of nanoparticles based on poly(isohexyl cyanoacrylate) that were prepared by anionic emulsion polymerization and were subsequently loaded with the anticancer drug doxorubicin. The success of the formulation is such that made it even into clinical trials for the treatment of liver cancer.^287,288^

**Figure 11:**
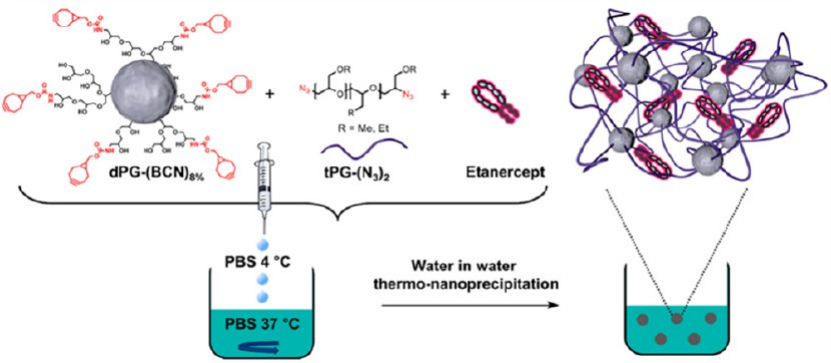
General scheme for the in situ encapsulation of proteins during the synthesis of nanogels via the nanoprecipitation methodology. dPG-(BCN)8% and tPG-(N_3_)2 refers to the polyglycerol-based building blocks that were used for the strain-promoted click chemistry. Reprinted with permission from ref (291). Open Access.

Besides the typical emulsion techniques, other methodologies have been developed by paying attention to the specific needs of the biomedical field. In a search for mild alternatives that enable the encapsulation and controlled release of sensitive cargoes like proteins, Calderón et al. adapted the nanoprecipitation methodology based on the ouzo effect,^289^ to synthesize soft nanoparticles using cold water as solvent and warm water as nonsolvent.^290^ The nanoprecipitation methodology to prepare polymer particles is based on the injection of concentrated polymer solutions into a nonsolvent for the polymer, with the requirement that both solvents are miscible with each other. Since thermoresponsive polymers have different solubility according to the temperature, they are ideal candidates to prepare polymer particles using the same solvent. Such approach was used to cross-link polyglycerol-based polymers via strain-promoted click chemistry, enabling the in situ encapsulation of the therapeutic protein Etanercept (Figure 11). The great potential of such protein laden nanogels for the treatment of inflammatory skin conditions was demonstrated using human skin and reconstructed skin models.^291^ This methodology was also used to prepare hybrid nanoparticles, using thermoresponsive polymers as the organic component and metal nanoparticles (Au, FeO, etc.) as the inorganic counterpart.^292,293^ The products have found applications in the novel upcoming field of theranostics, in which concepts of therapeutics and diagnostics are combined in a single particle entity.^294,295^

Latexes with nanometer and micrometer dimensions have also raised a great deal of interest in the area of diagnosis, as they can serve as platforms for a broad series of assays, with a higher sensitivity than that of the standard colorimetric methods. Very often, they have been combined with fluorescent dyes, thereby enabling their easy tracking upon mixing with complex biological fluids. They have been prepared with different polymer compositions, with polystyrene being the most popular polymer in commercial systems. They are typically used as tracers to elucidate biological events using fluorescence microscopy techniques and in immunoagglutination assays, for the detection of antibodies or antigens of interest. Very recently, they have been used for the development of tests for the detection of COVID-19 in thermometric lateral flow immunoassays.^296^

### 4.6 Polymer Colloids for Cosmetics and Personal Care

Colloidal particles produced by (mini)emulsion polymerization have had an important role for decades in cosmetic and personal care products. For example, microbeads, which are micron-sized particles based mainly on polyethylene (around 90% of the market), polystyrene, or poly(methyl methacrylate), were a very famous and efficient cosmetic ingredient used in toothpastes, facial and body scrubs, exfoliants, industrial hand cleaners, sunscreen lotion, and makeup.^297^ Their rigidity and size (c.a. 500 μm) conferred physical properties to exfoliate or cleanse in rinse-off for the skin, as they could rub-off make up and dead cells. Similarly, microgel particles, especially cross-linked poly(methacrylic acid) or poly(ethylene glycol)-based copolymer particles,^298^ were successful as delivery systems for cosmetic active ingredients. Also, recent design of multiresponsive microgels for delivery of actives have shown great potential.

However, the cosmetic and personal care market is dictated by a strong consumer perspective. The recent emergence of scientific evidence regarding the fate, persistence, and toxicity of plastic microbeads has attracted both public and regulatory concern about their widespread use and their potential for reaching the environment (in particular marine ecosystem), resulting in legislation restriction.^299,300^ While the UK already banned the use of microbeads for rinse-off products,^301^ the European parliament received draft regulation in September 2022 to be voted in the upcoming months to ban their use. In that sense, consumers are now very cautious with regard to the origin, sustainability, and degradability of such products. That is the reason why the effort to obtain a fully biodegradable alternative is crucial and emulsion polymerization-based products may have a commercial viability in this sector. Similarly, it is not only the core of the particles that must be taken into account. Poly(ethylene glycol) (PEG) is commonly used as a surfactant for emulsion stabilization, both in cosmetic formulations and for emulsion polymerization. Here again, cosmetic products containing PEG are not popular among consumers and, although they are not yet banned, cosmetic companies are preferring natural and biodegradable alternatives.^302^ In that sense, a recent development in polysaccharide-based emulsifiers is quite attractive to replace current oil-based emulsifiers.^303^ A slight modification of the natural polysaccharide with hydrophobic groups can confer interfacial activity to the natural polymer. In addition, amphiphilic block polypeptide-based emulsifiers have also been developed. Although, PEG is still used in the hydrophilic block, there are some alternatives such as polycarbonate,^304^ polysaccharides, and polyoxazolineoxoethylene.^305^

In summary, emulsion-based particles are facing an environmental challenge as currently most of the emulsion products are petroleum-based, and although mostly biocompatible, they are in general nonbiodegradable. The cosmetic and personal care sector have established some parameters, based on consumers preferences, that now dictate the new trends, like the biodegradability test, the naturality index (ISO 16128 standard), and the sustainability coefficient, the latter mainly led by L’Oréal.^306^ Thus, the work mentioned in section 3 on biodegradable polyesters produced by aqueous miniemulsion polymerization or the use of biobased monomers are crucial for the replacement of microbeads for cleanse applications. Waterborne colloids have already managed the reduction of volatile organic compounds, but the core of the particle and the surfactant used must be designed considering the environmental and sustainable aspect. Actually, one can assume that controlling the biodegradability of the colloidal particles would allow for the design of new delivery systems for cosmetic active ingredients. In addition, colloidal particles produced by emulsion polymerization could find application as Pickering emulsifiers, which is currently dominated by inorganic particles.^307^ Moreover, the correct design of the colloidal stabilizer is also expected to offer new generation of particles with biodegradable skin penetration enhancers, such as polysarcosine, that could allow one to increase the activity of the active ingredient drastically. Overall, the progress in obtaining more environmentally friendly colloidal particles by (mini)emulsion polymerization can be foreseen as the next generation of smart and targeted delivery systems to boost the efficacy of natural active ingredients.

## 5 Unconventional Ways of Processing Latexes

One of the primary reasons for the increased use of polymer colloids in applications such as coatings and adhesives in place of solvent-based systems is their unique viscosity profile. In the case of solution polymers of high molar mass, there is a limited regime in which viscosity remains at a workable limit. This requires the use of large amounts of solvent, which are subsequently released to the atmosphere. In contrast, colloidal systems are not affected by the molar mass of the polymer and relatively high solids content can be reached before there is a notable increase in viscosity. This advantage is now being recognized in new, alternative methods for the processing of polymer materials as detailed below.

### 5.1 Production of Advanced/Multifunctional Nanofibers by Electrospinning of Emulsion Polymers

Electrospinning is a well-established technology used to create polymer nanofibers. This technology has gained relevance in the last years due to its simplicity and low cost as well as the possibility to effectively scale up to industrial production.^308−312^ Electrospun nanofibers have exceptional properties such as a huge area/volume ratio, porous structure, and tunable functionality. These unique properties make electrospun materials very attractive for a broad range of applications such as textiles, filters, tissue engineering, drug delivery, wound healing, sensors, energy storage, catalysis, and many more.^313−317^

The traditional electrospinning process consists in feeding a polymer solution under a high applied electric field. When the force created by the voltage surpasses the resistance of the surface tension of the solution, a fine charged jet is ejected from the surface of the charged polymer solution toward the collector. On the way to the collector, the jet suffers a strong bending and elongation at the same time that the solvent evaporates, resulting in the deposition of a polymer nanofiber mat on the collecting electrode.

Solution electrospinning is the most widely used electrospinning method; however, it presents at least two serious limitations for its industrial application. The first limitation is the need to use toxic and flammable organic solvents, which can be problematic for industrial production due to the increasingly stringent environmental and safety regulations. Additionally, for many biorelated applications, such as tissue engineering or drug release, the toxicity of organic solvents could be highly critical. As an alternative, water can be used as a solvent, but in this case, only water-soluble polymers can be electrospun. In addition, the produced nanofiber material will also be water-soluble, a fact that will severely limit the number of applications. There are different cross-linking methods to increase the water resistance of water-soluble nanofibers, but they usually require high temperatures or toxic cross-linkers.^318−321^ The second limitation is related to the concentration of the electrospinning solution. There is a maximum critical concentration that can be used, which is around 10–15 wt % of the polymer. Polymer solutions of higher concentrations are not spinnable due to their high viscosity. This concentration limitation decreases the productivity of the electrospinning process significantly.

**Figure 12:**
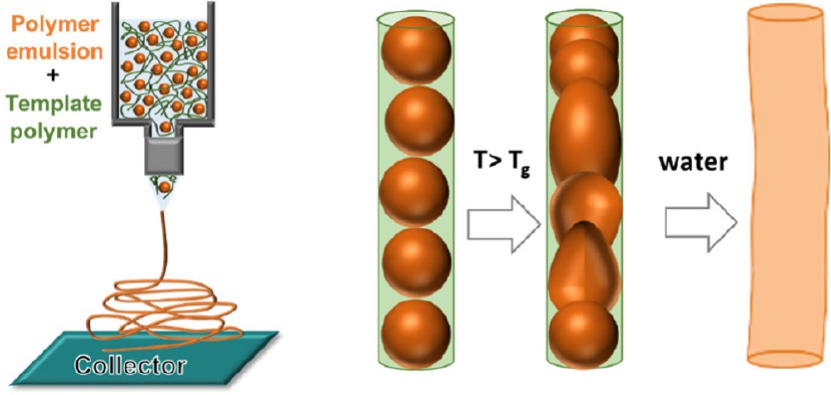
Diagram explaining the green electrospinning process and the obtained nanofibers.

Green electrospinning, is a novel method that consists in the use of latexes as electrospinning medium with the help of a template polymer (a water-soluble polymer). Green electrospinning overcomes the above-mentioned limitations, as it allows the use of water as electrospinning medium, even for hydrophobic polymers, and enables dispersions of higher polymer concentrations to be spun, increasing the overall productivity of the process.^322^ A template polymer is necessary to create chain entanglements and acts as a binder between the polymer particles during fiber formation. If desired, it can be removed at the end of the process by introducing the nanofiber mat in water (Figure 12).

The first paper on green electrospinning was published by Greiner and co-workers in 2007.^323^ Since then, different works have been published where diverse types of polymer particles have been electrospun, such as poly(styrene) (PS),^324−328^ poly(styrene-*co*-butyl acrylate) (P(S-*co*-BA),^329^ poly(butyl acrylate-*co*-methyl methacrylate) (P(BA-*co*-MMA) bearing cross-linkable monomers,^330,331^ waterborne polyurethanes (WPU), and more complex systems such as microgels,^332^ core–shell particles,^333−336^ or block copolymers.^337^ With regard to the type of template polymer, although poly(vinyl alcohol) (PVA) has been the most frequently used one, other water-soluble polymers such as poly(ethylene oxide) (PEO) or poly(vinyl formamide) (PVFA)^330^ have also been employed. These novel composite nanofibers obtained by green electrospinning have been claimed to have potential applications in tissue engineering, medicine, pharmacy, agriculture, and sensor technology.^332,333,336−338^

Although different template/particle ratios,^323,326,328,339^ particles with different sizes,^323,326,329^*T*_g_ values,^329^ or cross-linking densities^330,331^ have been spun, there are very few works in the literature that thoroughly study the influence of the initial dispersion composition on the final fiber morphology. To this end, Gonzalez et al.^340^ performed a systematic study that investigated the effect of the template polymer molar mass on the fiber quality, the total solids content of the dispersion, and the particle/template ratio in a single work. They also investigated for the first time, the influence of the surfactant used to stabilize the polymer particles, the surface functionality of the polymer particles, and the use of a bimodal particle size distribution. They demonstrated that all these parameters affect the viscosity of the initial complex dispersion and therefore have a strong influence on the final fiber morphology and that a minimum viscosity was needed to produce good quality fibers without defects. This work highlights the importance of the emulsion polymerization process and the potential of tuning polymer colloids for electrospinning applications. As a conclusion, green electrospinning is a promising technology to achieve clean and safely scalable electrospun nanofibers with unique properties that could not be obtained by any other method.

### 5.2 Additive Manufacturing of Waterborne Latexes: A Platform for Vat Photopolymerization of High Molar Mass Polymers

Additive manufacturing (AM) modalities employ a wide range of polymers that each offer unique advantages and disadvantages. In general (pre)polymer solutions or bulk polymer materials are used as printing materials, but some researchers have investigated latexes as viable precursors for various AM processes, primarily leveraging the higher viscosity regime accessible in direct ink write (DIW) processes.^341,342^ Two AM platforms have emerged as the most promising for printing polymeric latexes: DIW and vat photopolymerization (VP). Traditionally, DIW AM employs high viscosity filled resins at the expense of resolution, whereas VP imparts micron-scale resolution with viscosity limitations. Reducing the limitations of an increased viscosity for a high molar mass polymer, Long et al. combined silica and a synthetic latex to provide structural integrity together with ultraviolet-assisted direct ink write (UV-DIW) AM.^343^ Styrene–butadiene rubber (SBR) latex, at a constant solids content in water, free radical photoinitiator, n-vinylpyrrolidone (NVP), and poly(ethylene glycol) diacrylate (PEGDA) as water-soluble scaffold precursors provided a photocurable colloidal dispersion. The silica content was systematically varied from 0% to 50% silica (0:100 to 50:50 silica:SBR) while keeping the solids content of the SBR constant. These formulations exhibited rapid reversible modulus crossovers at low (0.1%) and high (50%) strain amplitude, indicating a reversible liquid–solid transition. Furthermore, shear-thinning behavior typical of DIW dispersed systems with the 50:50 system exhibiting 3–4 Pa s viscosities at high shear rates (10^3^ s^–1^). Printed and cast samples (30:70 silica:SBR) showed comparable strains at break of 320% and 317%, respectively. However, the printed samples showed lower average ultimate strength as compared to cast samples, 7.6 and 8.2 MPa, respectively, perhaps as a result of imperfections or layering introduced through the printing process. In contrast, VP provides a platform for addressing these limitations due to the introduction of UV curing that enables isotropic properties.

Vat photopolymerization takes advantage of UV curable functionality, e.g., acrylates, to enable layer-by-layer curing of a resin within a vat.^344−346^ Resin viscosity becomes the primary limiting factor with high viscosity resins not sufficiently recoating the build platform prior to printing the next layer. Recoating blades provide solutions for specific resin types and viscosity ranges but typically remain limited in the use of a wide range of materials while also reducing the speed of the printing process.^347,348^ Addressing the commonplace limitations of VP reactive formulations in a novel and innovative way will continue to drive the technology forward. Latex provides a novel approach to addressing these platform limitations, providing high molar mass materials at viscosities well below the upper limit accessible to VP (>10 Pa s).^343^ Due to the biphasic nature of the latexes, the polymer chains are sequestered to their own dispersed phase surrounded by water, resulting in viscosities of 1 Pa s or below. Water-soluble reactive diluents provide a means for achieving sufficient moduli to maintain structural fidelity throughout the printing process, enabling the printing of complex geometries. However, upon exposure, due to the presence of latex particles dispersed in water, light scattering occurs that must be compensated to achieve the resolution expected of VP. Computer-vision-based processing parameter generation developed by Williams et al. has proven successful in compensating for the light scattering characteristic of colloidal dispersions.^349^

**Figure 13:**
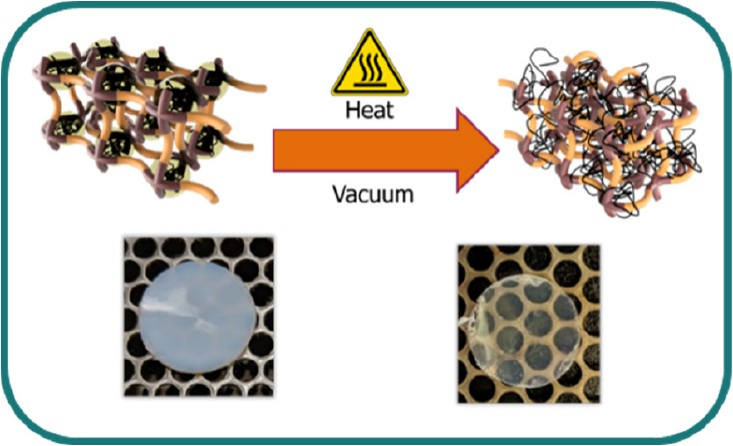
Graphical depiction of the drying and coalescence process of photocured greenbody to form a sIPN. Reprinted from ref (350). Copyright 2020 American Chemical Society.

The combination of photoreactive latex formulations and computer-vision-based processing parameter generation yielded parts that exhibited isotropic properties while having useable viscosities for VP. Using water-soluble free radical photoinitiators with NVP and PEGDA as scaffold precursors, Long et al. produced green bodies with entrapped SBR latex particles (Figure 13).^350^ These reactive diluents ensured sufficient modulus (classically ranging from 10^4^–10^6^ Pa) to maintain feature fidelity throughout the printing process. Upon greenbody formation, subsequent annealing above the *T*_g_ of the latex promoted particle coalescence, forming a semi-interpenetrating polymer network (sIPN), ensuring the properties of the previously dispersed high molar mass polymer (Figure 13). Tensile testing of printed SBR latex, a material previously inaccessible to VP, resulted in precise geometries exhibiting extensibilities exceeding 500%. Furthermore, dynamic mechanical analysis (DMA) indicated sIPN formation through the presence of a single *T*_g_, which was corroborated by transmission electron microscopy (TEM). Printing these novel formulations provided a versatile platform for the VP of various waterborne high molar mass polymers. Further expansion on VP accessible materials will provide an excellent opportunity for achieving complex geometries with isotropic properties and excellent resolution enabling rapid processing of multifaceted materials. These works clearly show the potential of using polymer colloids in additive manufacturing technology.

## 6 Conclusions and Outlook

The global market for polymer colloids continues to increase and shows little signs of slowing in the coming years. In fact, the use of water as dispersing media makes it well suited to face many of the current sustainability issues faced by the polymer industry. Furthermore, as detailed in the latter parts of this perspective, the possibility to control and synthesize materials with multiple characteristics makes polymer colloids well-suited to many emerging applications.

That said, it is clear there is still much to be done. From a fundamental point of view there are still many aspects of the emulsion polymerization process itself that remain puzzling and hard to understand in a quantitative way. These features are largely related to events that occur in the aqueous phase, which control the rate of radical entry and exit from particles as well as nucleation phenomena. From a production point of view, we are also often left trying to control a process in which we often have no method for online measurement and limited control options even if perfect knowledge of the current reactor state were available. One way to improve the production efficiency at the industrial scale would therefore be the development of in-line sensors for particle size and molar mass distributions or through mathematical models that can be used as soft sensors for control purposes. From a products point of view, there are also many ongoing challenges as a result of the constantly shifting commercial landscape where there is a continuing drive to decrease VOC content of latex systems in their major market applications.

The success of emulsion polymerization is also contributing to exacerbating some of the major challenges currently faced by industry in that proposed changes to the established technologies require them to be cost-competitive with processes that have been continually developed over the course of a century. This is particularly the case with the necessary transition away from petro-chemical sourced feedstocks with biobased alternatives. While undoubtedly desirable from a consumer point-of-view, product standards need to keep at the same level, meaning that the final properties (and price) of the product should be the same. As mentioned in the section of the biobased materials, this transition has been slower than predicted from an industrial point of view. Thus, efforts must be directed to find renewable materials at scale, and to create sustainable synthesis and polymerization routes to improve the sustainability of the whole processes. One way to improve the sustainability of coatings and adhesives is the synthesis of (bio)degradable polymer materials, and as has been mentioned, this route offers different possibilities to incorporate the degradable groups in the polymeric chain.

Both in established applications, such as coatings and adhesives, and in new applications the physical properties of colloidal particles are a major driver in their commercial development. The high specific surface area of colloidal systems in particular has been demonstrated to be a major advantage in energy applications as well as in carbon capture technologies. In addition, one of the primary benefits of using colloidal polymer is related to the use of water as a dispersing media and the ability to produce dispersions with high molar mass at low viscosity. This has long been acknowledged in the coatings market but is now being utilized in new applications such as green electrospinning and 3D printing, which in the future can leverage much of the knowledge that has already been generated for use of colloidal particles in other applications.

Overall, with the advances underlined in this work, we would like to reaffirm the potential of polymer colloids in a diverse range of systems. Emulsion polymerization is capable of producing an amazing range of colloidal structures with excellent control over the polymer properties. We firmly believe that this flexibility can promote the use of emulsion polymers not only in the traditional markets of coatings and adhesives but also can offer a solution to some of the most pressing societal challenges related to energy and sustainability.
